# The Effects of Ritonavir on the Pharmacokinetics of Tofacitinib in Rats

**DOI:** 10.3390/ph18101561

**Published:** 2025-10-16

**Authors:** Sung-yoon Yang, Hyunjung Lee, Tham Thi Bui, Quyen Thi Tran, Lien Thi Ngo, Hwi-yeol Yun, Sangkeun Jung, Jung-woo Chae

**Affiliations:** 1 College of Pharmacy, Chungnam National University, Daejeon 34134, Republic of Koreabttham@hpmu.edu.vn (T.T.B.); hyyun@cnu.ac.kr (H.-y.Y.); 2 Department of Bio-AI Convergence, Chungnam National University, Daejeon 34134, Republic of Korea; hjung0222@gmail.com; 3 Faculty of Pharmacy, Haiphong University of Medicine and Pharmacy, Haiphong 04212, Vietnam; 4 Faculty of Pharmacy, PHENIKAA University, Yen Nghia, Ha Dong, Hanoi 12116, Vietnam; quyen.tranthi@phenikaa-uni.edu.vn (Q.T.T.); lien.ngothi@phenikaa-uni.edu.vn (L.T.N.); 5 A&A Green Phoenix Group JSC, PHENIKAA Research and Technology Institute (PRATI), No. 167 Hoang Ngan, Trung Hoa, Cau Giay, Hanoi 11313, Vietnam; 6 Senior Health Convergence Research Center, Chungnam National University, Daejeon 34134, Republic of Korea; 7 Department of Computer Science and Engineering, Chungnam National University, Daejeon 34134, Republic of Korea

**Keywords:** tofacitinib, ritonavir, pharmacokinetics, drug–drug interaction, CYP3A4

## Abstract

**Background and Objective**: Tofacitinib (TOF), an oral Janus kinase inhibitor used to treat rheumatoid arthritis (RA), is extensively metabolized by cytochrome P450 (CYP) 3A4. Ritonavir (RTV), a protease inhibitor, is commonly used as a pharmacokinetic (PK) enhancer due to its potent CYP3A4 inhibitory effects. Considering the prevalence of comorbidities in RA patients, it is possible to use TOF and RTV concurrently, raising concerns about potential drug–drug interactions (DDIs). The current study aims to assess the potential DDIs between RTV and TOF. **Methods**: An in vivo rat study was conducted to investigate the impacts of RTV on the PK of TOF. Rats were randomly divided into three groups: vehicle, RTV 10 mg/kg, and RTV 20 mg/kg, each undergoing four days of pretreatment. On the test day, TOF (10 mg/kg) was administered following co-administration of the respective RTV doses. Blood samples were collected at the pre-specified time points. Plasma concentrations of TOF were quantified using liquid chromatography coupled with mass spectrometry, and PK parameters were analyzed using non-compartmental analysis. **Results**: RTV (10 and 20 mg/kg) increased the area under the curve of TOF by 2.53-fold (95% CI: 1.64–3.43) and 5.39-fold (95% CI: 4.47–6.33), respectively, and the maximum concentration by 1.47-fold (95% CI: 0.99–2.00) and 2.86-fold (95% CI: 2.39–3.37), respectively. Whereas the half-life (t_1/2_) remained unchanged. **Conclusions**: RTV substantially increased TOF exposure in rats. These results suggest the need for dose adjustments of TOF during co-administration with RTV in clinical settings. Further clinical research is needed to confirm these findings.

## 1. Introduction

Tofacitinib (TOF), a novel oral Janus kinase inhibitor [1], has emerged as a treatment option for rheumatoid arthritis (RA), psoriatic arthritis, and ulcerative colitis [2,3]. The clinical pharmacokinetics (PKs) of TOF show dose-proportional characteristics with rapid absorption and elimination [4]. Renal clearance accounts for 30% of the total clearance, whereas the remaining 70% is attributed to hepatic clearance. Notably, TOF is predominantly metabolized by the cytochrome P450 3A4 enzyme (CYP3A4), accounting for 50 to 55% of the overall clearance, with CYP2C19 playing a minor role in this process [5]. Tofacitinib is metabolized into 29 identified metabolites (M1-M29) via CYP3A4/5-mediated oxidation, N-demethylation, and glucuronidation, with all human metabolites detected in at least one preclinical species. These metabolites are predicted to have ≤10-fold the potency of the parent drug for Janus kinase (JAK) 1/3 inhibition, suggesting they are likely pharmacologically inactive; however, empirical in vitro data or metabolite-specific pharmacokinetic profiles are currently unavailable to confirm their activity [6]. This metabolic profile suggests that the co-administration of TOF with CYP inducers or inhibitors could potentially influence its PKs. A clinical drug interaction study demonstrated that fluconazole (a moderate CYP3A4 inhibitor and strong CYP2C19 inhibitor) and ketoconazole (a strong CYP3A4 inhibitor) increased the area under the concentration–time curve (AUC) of TOF by 79% and 103%, respectively, in healthy human subjects [7].

Ritonavir (RTV), a strong CYP34 inhibitor, is strategically co-administered with protease inhibitors for human immunodeficiency virus (HIV) treatment or with antiviral agents like nirmatrelvir for COVID-19 treatment to increase the exposure of CYP3A4 substrates, leading to enhanced therapeutic efficacy [8,9,10]. However, RTV can also inadvertently increase exposure to other CYP3A4 substrates when co-administered [11], potentially leading to unwanted drug–drug interactions (DDIs). Tacrolimus exposure was reported to increase to toxic levels within 3 days of co-administration with RTV in patients undergoing long-term tacrolimus treatment [12]. In another study, oxycodone exposure tripled in the presence of RTV, leading to adverse events such as respiratory depression [13]. Furthermore, RTV moderately induced CYP2C19 activity; co-administration of RTV at a dose of 400 mg twice daily substantially decreased steady-state AUC of voriconazole, a substrate of CYP2C19, by 82% and the maximum concentration of TOF (C_max_) by 66% in healthy subjects [14].

Tofacitinib is used for treating RA and ulcerative colitis (UC), with RA affecting approximately 1–2% of the global population [15] and UC having a prevalence of 0.5% in developed countries [16]. Tofacitinib is prescribed in 10–15% of RA cases, particularly for patients unresponsive to conventional therapies. Ritonavir is widely used in HIV treatment, with an estimated 38 million people living with HIV globally [17]. The co-administration of tofacitinib and ritonavir is of interest due to potential pharmacokinetic interactions in patients with comorbidities, such as those with both RA and HIV. Considering the prevalence of multimorbidity under rheumatic conditions, it is possible that RTV and TOF could be used concurrently [18,19]. The co-administration of these two drugs may raise concerns about potential DDIs, leading to unwanted clinical outcomes ranging from therapeutic failures to adverse events. Ytterberg et al. [20] recently raised concerns about the safety profile of TOF, noting an increased incidence of pulmonary embolism and higher mortality among patients receiving a dose of 10 mg TOF (BID) compared with those receiving 5 mg TOF (BID). This suggests that TOF increased the risk of adverse events in a dose-dependent manner. Therefore, assessing the DDIs between TOF and RTV is imperative given the association of higher TOF exposure with an increased safety risk.

This study aimed to assess the PK DDIs between TOF and RTV in a rat model, providing a foundation for understanding potential clinical implications. The plasma TOF concentrations were quantified using a validated analytical bioassay, and non-compartmental analysis (NCA) was employed to calculate PK parameters. Statistical analysis was used to evaluate potential DDIs, with findings intended to guide future human studies.

## 2. Results

### PK DDI Evaluation

**Figure 1:**
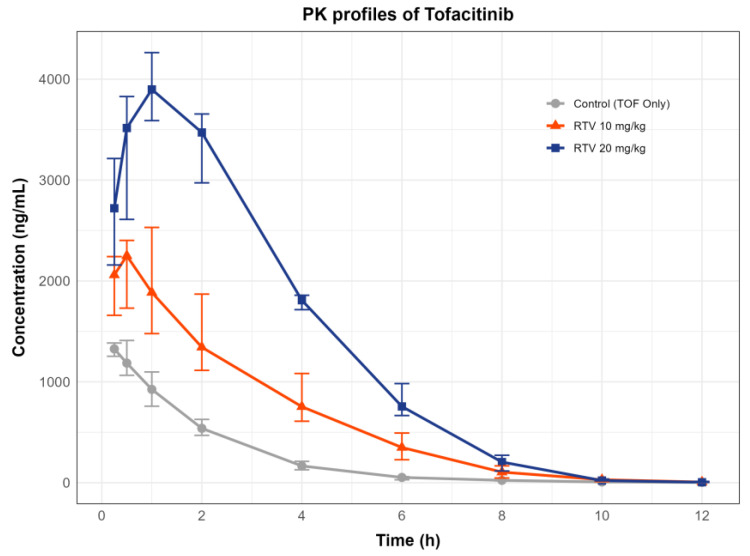
Observed plasma concentration-time profiles of TOF following the oral administration of TOF with or without RTV pretreatment in rats. Each point and bar represent the median and the interquartile range of the TOF concentrations. Control (TOF only): coadministered with vehicle, RTV 10 mg/kg: coadministered with 10 mg/kg of RTV, and RTV 20 mg/kg: coadministered with 20 mg/kg of RTV. The elevated concentration at 0.25 h reflects rapid absorption post-dosing, amplified by RTV pretreatment and CYP3A4 inhibition, consistent with the observed Cmax values. The 0 h time point is omitted (pre-dose levels undetectable, see Section 3).

The observed concentrations of TOF versus time in the rat plasma were quantified using a validated LC-MS/MS method. Figure 1 shows the PK profiles of TOF in the experimental groups. At 12 h, the mean concentrations were 21.9 ng/mL (RTV 10 mg/kg) and 12.1 ng/mL (RTV 20 mg/kg), with the lowest values at 1.5 ng/mL and 2.6 ng/mL, respectively, all exceeding the LLOQ of 1 ng/mL.

**Figure 2:**
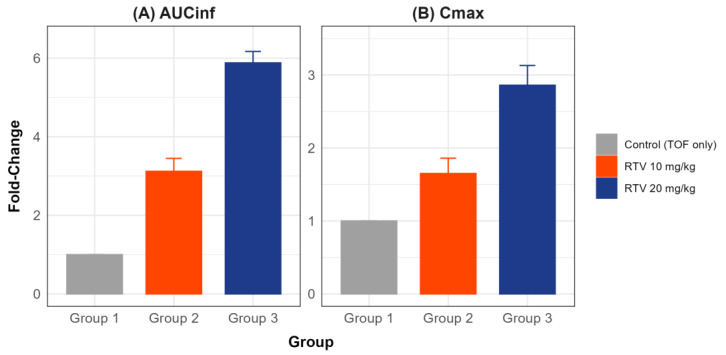
Fold-changes in pharmacokinetic parameters of TOF across experiment groups, relative to the Control (Vehicle) group. (**A**) Fold-change in AUC_inf_ and (**B**) fold-change in Cmax. Error bars represent the 95% confidence intervals of the mean fold change, calculated using bootstrap resampling. Group 1: Control (TOF only), Group 2: RTV 10 mg/kg, and Group 3: RTV 20 mg/kg.

NCA was performed to calculate the PK parameters of TOF, with and without RTV (Table 1). Statistical comparisons using the Kruskal–Wallis test revealed significant differences in the AUC_inf_, C_max_, and T_max_ between the groups (*p* < 0.01), whereas t_1/2_ remained unchanged (*p* = 0.08). The observed significant differences in AUC_inf_ and C_max_ (*p* < 0.001) confirm that the sample size of n = 8 per group provided adequate statistical power for the primary endpoints, as justified by a priori power calculations. AUC_inf_ increased 2.53-fold (95% CI: 1.64–3.43) in RTV 10 mg/kg and 5.39-fold (95% CI: 4.47–6.33) in RTV 20 mg/kg compared with that in Control (TOF only), with post hoc analysis confirming significant differences across all groups (*p* < 0.016). C_max_ increased 1.47-fold (95% CI: 0.99–2.00) in RTV 10 mg/kg and 2.86-fold (95% CI: 2.39–3.37) in RTV 20 mg/kg, showing a significant difference between Groups 1 and 3 and between RTV 10 mg/kg and RTV 20 mg/kg. Notably, T_max_ in RTV 10 mg/kg showed high variability, likely due to inter-individual differences in gastrointestinal absorption. The bioanalytical method was validated with QC samples, demonstrating high accuracy and precision, as detailed in the Methods section, ensuring reliable quantification of TOF concentrations. Figure 2 illustrates the fold-changes in PK parameters of TOF across experimental groups relative to the Control group, presented as bar charts. for AUC_inf_ shows an approximate 3-fold increase for Group 2 and a 6-fold increase for Group 3 compared to Group 1, which remains at approximately 1-fold. C_max_ exhibits an approximate 2-fold increase for Group 2 and a 3-fold increase for Group 3, with Group 1 at approximately 1-fold. The median clearance values are 3.57 L/h/kg [2.65–4.49] for the Control (TOF only) group, 1.65 L/h/kg [1.19–2.11] for the RTV 10 mg/kg group (0.46-fold relative to Control, 95% CI: 0.33–0.59), and 0.66 L/h/kg [0.60–0.73] for the RTV 20 mg/kg group (0.18-fold relative to Control, 95% CI: 0.13–0.23), with a statistically significant difference across groups (Kruskal–Wallis test, *p* < 0.001).

## 3. Discussion

The rapid rise in tofacitinib concentration at 0.25 h (Figure 1) aligns with its established rapid absorption profile [4], with RTV pretreatment enhancing exposure through CYP3A4 inhibition, as reflected in the increased AUCinf and Cmax (Table 1). This study provides preclinical evidence of significant pharmacokinetic drug–drug interactions (DDIs) between tofacitinib (TOF) and ritonavir (RTV), primarily driven by CYP3A-mediated pathways. These findings highlight the potential need for dose adjustments in clinical settings to mitigate DDI risks, particularly for patients with comorbidities requiring concurrent TOF and RTV use. The CYP3A4-mediated pathway plays a major role in DDIs between TOF and RTV as TOF is primarily metabolized by CYP3A4. An in vitro study demonstrated that ketoconazole inhibits over 70% of the overall metabolism of TOF [5]. This aligns with the findings of a phase 1 clinical trial, in which co-administration of ketoconazole led to an increase in the AUC and C_max_ of TOF by 103% and 16%, respectively [7]. RTV is a potent CYP3A4 inhibitor that acts in both a time- and dose-dependent manner [21]. This potency is supported by Rioux et al. [22], who demonstrated that ritonavir inhibited midazolam hydroxylation with an IC50 of ~11 nM in rat liver microsomes, confirming potent CYP3A inhibition in vitro. Similarly, Kirby et al. [23] demonstrated that ritonavir significantly reduces intestinal CYP3A4 activity in humans, leading to a profound increase in the oral exposure of probe substrates like midazolam, providing a direct clinical parallel to our preclinical observations with tofacitinib. To complement our in vivo findings, we draw on Lee et al. (2021), who reported significant inhibition of rat CYP3A1/2 and CYP2C11 by voriconazole in rat hepatic microsomes, suggesting that RTV likely exerts a comparable inhibitory effect on TOF metabolism in our study [24]. Additionally, immunoinhibition studies revealed that a polyclonal anti-rat CYP3A2 antibody inhibited midazolam (MDZ) metabolism by 80–90% in rat, human, and cDNA-expressed human CYP3A4 microsomes, indicating the critical role of the CYP3A4 subfamily in drug metabolism across species [25]. These findings support the hypothesis that RTV’s inhibition of CYP3A1/2 in rats, analogous to human CYP3A4, drives the observed increase in TOF exposure. Several clinical studies have indicated that RTV substantially affects exposure to CYP3A4 substrate drugs, exhibiting inhibitory potency comparable to or greater than that of ketoconazole [26]. Additionally, the CYP2C19-mediated pathway contributes to TOF metabolism, although to a lesser extent than CYP3A4. Fluconazole increases the AUC and C_max_ of TOF by 79% and 27%, respectively [7]. As a mild inducer of CYP2C19, RTV may also modulate TOF metabolism via this pathway, though its predominant effect is likely through CYP3A inhibition. To address potential protein binding effects, we refer to FDA data indicating that the unbound fraction of TOF in rat plasma increases in a concentration-dependent manner, whereas it remains relatively stable in human plasma [27]. This species-specific difference in protein binding could influence the free drug concentrations available for metabolism, potentially amplifying the DDI in rats compared to humans. While direct measurements of hepatic and intestinal RTV concentrations were not conducted, the potent CYP3A inhibitory effect of RTV, as evidenced by prior studies, likely results in elevated TOF concentrations in these tissues, contributing to the observed PK changes [28]. Further in vitro experiments, including tissue concentration analyses, could elucidate the precise contributions of CYP3A1/2 to the ritonavir-tofacitinib DDI.

The animal PK DDI study involved the use of a rat model to investigate the effect of RTV on TOF PKs, given the metabolic similarities of TOF in rats and humans [29]. Rat CYP3A1 shares approximately 73% homology with human CYP3A4 [30], and rat CYP2C11 is highly analogous to human CYP2C19. CYP3A1/2 and CYP2C11 are the main metabolizing enzymes abundant in the rat liver and intestine [31,32]. Several studies indicated that CYP3A1/2 and CYP2C11 are the main CYP450 enzymes related to TOF metabolism in a rat model; a 46% increase in AUC was observed in animals pretreated with ketoconazole, an inhibitor of CYP3A1/2, and a 39% increase in AUC in those pretreated with fluconazole, an inhibitor of CYP2C11 [33]. Additionally, a 166% increase in AUC was seen with voriconazole, which inhibits both CYP3A1/2 and CYP2C11 [24]. This suggests that the rat model is a viable option for assessing the DDI potential of TOF mediated by CYP450 pathways.

The study design reflected clinical settings, with RTV doses (10 and 20 mg/kg) set using body surface area normalization, equivalent to human doses of 100 and 200 mg [34]. The 4-day RTV pretreatment was selected to ensure steady-state CYP3A inhibition, as studies show that RTV reaches maximum inhibitory effects within 2–3 days in rats, and humans [35,36,37,38,39,40]. However, interspecies metabolic differences may affect the duration required, warranting validation in human-relevant models to enhance translatability. The TOF dose for rats was set at 10 mg/kg based on the dosing regimens used in previous PK studies [24,27,41,42,43,44,45], equivalent to 100 mg in humans, which is notably higher than the maintenance doses of TOF (5 mg BID or 10 mg BID). However, TOF exhibits linear kinetics within the range of 3–100 mg in humans [46], suggesting that the experimental setup can reflect the clinical conditions under which TOF and RTV can be co-administered.

RTV significantly increased TOF exposure in rats in a dose-dependent manner. RTV’s CYP3A inhibition significantly increased TOF exposure, likely due to reduced intestinal first-pass metabolism, as evidenced by the elevated AUC_inf_ and Cmax (Table 1). The observed variability in T_max_, particularly with RTV 10 mg/kg, may reflect inter-individual differences in gastrointestinal absorption, potentially influenced by ritonavir’s effect on gastric motility in male rats, consistent with prior studies [47,48]. In contrast, RTV 20 mg/kg showed less variability (T_max_, 1.0 [1.0–1.0] h), suggesting a more consistent effect on gastrointestinal absorption at a higher dose. The unchanged t_1/2_ suggests that hepatic clearance was maintained, potentially due to a balance between CYP3A1/2 inhibition and mild CYP2C11 induction in male rats, where CYP2C11 expression is prominent [32]. This implies that RTV’s primary effect is on intestinal first-pass metabolism, reducing TOF metabolism and increasing bioavailability. In rats, TOF exhibited a low bioavailability (BA) of 29.1%, primarily because of extensive intestinal first-pass effect (46.1%) and additional hepatic first-pass effect (21.3%) [41]. This supports the hypothesis that RTV-mediated CYP3A inhibition in the small intestine could increase TOF exposure by reducing first-pass metabolism. Co-administration of RTV with TOF warrants caution, and adjustments may be required to ensure patient safety.

Despite these insights, this study has several limitations. Inherent species differences, including physiological variations and differences in protein expression and enzymatic activities, may undermine the fidelity of human DDI predictions based on this study. The human BA of TOF is approximately 74%, which is higher than that in rats (29.1%), suggesting that first-pass metabolism of TOF in humans is less extensive than in rats. This study used a single TOF does (10 mg/kg), limiting the evaluation of the RTV effect’s linearity; future studies with a broader range of TOF does could assess does-dependent interactions and improve generalizability to clinical scenarios [41,46]. To complement our in vivo findings, in vitro studies, such as those by Lee et al. (2021), demonstrated significant CYP3A1/2 and CYP2C11 inhibition in rats with voriconazole, suggesting similar CYP-mediated mechanisms drive the RTV-TOF DDI [24]. Further studies in vitro assays (e.g., non-competitive inhibition, enzyme activity measurements in rat or human liver and intestinal microsomes, protein binding, or RTV tissue concentration studies) could clarify the contributions of CYP3A1/2 and CYP2C11 to this DDI, deepening mechanistic understanding [5,24].

The rat model used in this study provides robust preclinical evidence of the pharmacokinetic drug–drug interactions (DDI) between TOF and RTV, primarily through CYP3A-mediate pathways. Rat CYP3A4, and rat CYP2C11 is analogous to human CYP2C19, making the rat a suitable model for studying CYP-mediated DDIs [30,32]. The significant increases in TOF’s AUC_inf_ (2.69- and 5.31-fold) and Cmax (1.58- and 2.87-fold) with RTV co-administration align with RTV’s potent inhibition of CYP3A4 in humans, as observed with other CYP3A4 substrates such as tacrolimus and oxycodone [12,13]. These findings suggest that RTV increases TOF exposure in rats by reducing intestinal first-pass metabolism, a major contributor to TOF’s low bioavailability in rats (29.1%) compared to humans (74%), though species differences may alter DDI magnitude in humans [41]. In clinical settings, RA patients with comorbidities such as HIV or COVID-19 may receive RTV [9,10,18,19]. The dose-dependent increase in TOF exposure indicates a need for dose adjustments to mitigate risks of adverse events, such as pulmonary embolism, which are associated with higher TOF doses [20]. However, species differences in bioavailability and enzyme expression may result in quantitative differences in DDI magnitude between rats and humans. To address this, clinical pharmacokinetic studies in humans are warranted to confirm the extent of the DDI and establish precise dosing guidelines for safe co-administration of TOF and RTV.

This study reveals that RTV significantly enhances TOF exposure in rats, highlighting the potential need for dose adjustments in clinical settings to mitigate DDI risk [20]. While our study did not include chronic dosing, the mechanism-based inhibition of ritonavir, which causes irreversible enzyme inactivation [22], suggests that its inhibitory effect is likely to be durable over time. We acknowledge this as a limitation and an important direction for future research, particularly to assess the potential for long-term DDI effects and cumulative toxicity. Further clinical investigations are essential to these findings and establish precise dosing guidelines for the safe co-administration TOF and RTV. Additionally, the application of PBPK modeling could enhance the predictive power for clinical outcomes, enabling more precise translational research and practical clinical implementation. To enhance the translatability of rat PK data to humans, physiologically based pharmacokinetic (PBPK) modeling can be employed to account for species-specific differences, such as the lower bioavailability of tofacitinib in rats (29.1%) compared to humans (74%) and variations in CYP3A1/2 and CYP3A4 activity, thereby improving the prediction of clinical DDI outcomes.

## 4. Materials and Methods

### 4.1. Chemicals and Reagents

Tofacitinib citrate, RTV, and domperidone (DOMP) as an internal standard for the bioassay, were purchased from Sigma-Aldrich (Saint Louis, MO, USA). Domperidone was selected as the internal standard due to its chemical stability, appropriate ionization behavior under ESI conditions, distinct mass spectrometry signals that do not interfere with TOF detection, and suitable chromatographic retention time for reliable quantification. Formic acid was obtained from SAMCHUN pure chemical Company (Pyeongtaek, Gyeonggi-do, Republic of Korea). High-performance liquid chromatography-grade methanol and water were obtained from Thermo Fisher Scientific (Waltham, MA, USA).

### 4.2. Animals

The animal study was conducted in accordance with all the relevant guidelines and regulations of the Institutional Animal Care and Use Committee (IACUC) of Chungnam National University (approval number: 202304A-CNU-064). Seven-week-old male Sprague–Dawley rats (*n* = 8 per group) were obtained from Orient Bio, Inc. (Seongnam, Gyeonggi-do, Republic of Korea). The mean body weights of the rats were 252.0 g for Group 1, 252.7 g for Group 2, and 253.3 g for Group 3, measured at the start of the experiment (n = 8 per group). The animals were housed in a controlled environment maintained at 23.5 ± 0.5 °C, 53.5 ± 2.8% humidity, and a 12 h light/dark cycle, with free access to a standard diet and water.

### 4.3. In Vivo PK DDI Study

**Figure 3:**
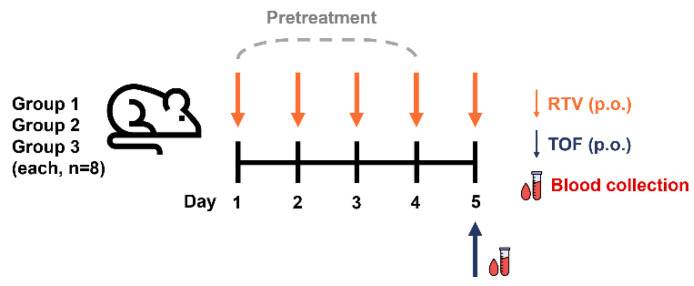
Schematic of the preclinical PK DDI study. Rats were pretreated with RTV for four consecutive days (Group 1: Control (TOF only); Group 2: RTV 10 mg/kg; Group 3: RTV 20 mg/kg). On the test day (Day 5), each rat received 10 mg/kg of TOF at 30 min after the relative RTV dose. Blood samples were collected at the specified time points.

An in vivo rat study was conducted to assess PK DDIs between TOF and RTV. Figure 3 shows a schematic diagram of the PK-DDI study. The rats were randomly divided into three groups (eight rats per group) after 1 week of acclimatization. Each rat was pretreated with RTV by oral gavage at different doses for four consecutive days (from Day 1 to Day 4) to ensure steady-state CYP3A inhibition, based on studies indicating rapid onset of inhibition [35,36]. Group 1 (control group) received the vehicle (normal saline). Groups 2 and 3 served as test groups and received 10 and 20 mg/kg RTV once daily (QD), respectively. On the next day (Day 5), rats from all groups were administered TOF (10 mg/kg) via oral gavage 30 min after the RTV dose. Serial blood samples (150 μL) were collected from the tail vein into EDTA tubes before dosing and 0.25, 0.5, 1, 2, 4, 6, 8, 10, and 12 h after TOF administration. The collected samples were centrifuged at 10,000 rpm for 10 min at 4 °C to separate the plasma and stored at −80 °C until analysis. Ritonavir plasma concentrations were not measured, as the study aimed to characterize changes in tofacitinib exposure as the victim substrate in this DDI; this is consistent with numerous DDI studies that focus on substrate pharmacokinetics to evaluate interaction magnitude (e.g., midazolam with itraconazole [49,50], probe substrates in CYP inhibition assessments [51,52]).

### 4.4. LC-MS/MS Method for Quantification of TOF in Rat Plasma

Plasma concentrations of tofacitinib (TOF) were quantified using a validated liquid chromatography mass spectrometry (LC-MS/MS) method. Plasma samples were prepared by protein precipitation with acetonitrile containing domperidone as an internal standard (100 ng/mL). After vortex mixing and centrifugation at 15,000× *g* for 10 min at 4 °C, the supernatant was analyzed by LC-MS/MS with an injection volume of 5 μL. Chromatographic separation was achieved on a C18 reversed-phase column with a mobile phase of 0.1% formic acid in water and acetonitrile at a flow rate of 0.4 mL/min. Mass spectrometry was performed in positive ion mode with an electrospray ionization probe. The calibration curve was linear over 1–5000 ng/mL (R^2^ > 0.99). Quality control samples, analyzed in triplicate, met FDA/EMA bioanalytical validation criteria for accuracy and precision [53,54]. Detailed instrument settings, sample preparation, calibration, and validation data are provided in Supplementary Material (Tables S1 and S2; Figure S1). Figure S1 includes representative chromatograms with labeled peaks for tofacitinib (4.25 min) and domperidone (IS, 4.56 min), updated to reflect the method’s validation accuracy.

### 4.5. Pharmacokinetic Evaluation

NCA was performed using the NonCompart package (version 0.7.0) in R to calculate the following PK parameters of TOF: AUC from zero to infinity (AUC_inf_), C_max_, time to reach maximum concentration (T_max_), and half-life (t_1/2_).

### 4.6. Statistical Analysis

Statistical analyses were performed using R software (version 4.4.1). The pharmacokinetic (PK) parameters exhibited a non-normal distribution, as confirmed by the Shapiro–Wilk test (*p* < 0.05), due to the small sample size (*n* = 8 per group) and the inherent variability in PK data. Therefore, the Kruskal–Wallis test, a non-parametric method, was chosen to compare medians across multiple groups without assuming normality or equal variances. The Kruskal–Wallis (K–W) test was employed, followed by post hoc analysis with the Bonferroni correction. To account for multiple comparisons across the three groups, the Bonferroni correction was applied, adjusting the significance level to *p* < 0.016 (0.05/3) to reduce the risk of Type I errors. Statistical significance for the K–W test and post hoc analysis was set at *p* < 0.05 and *p* < 0.016, respectively.

## 5. Conclusions

Herein, we assessed the effects of RTV on the PKs of TOF. The insights gained from this study may contribute to a better understanding of the PK DDIs between RTV and TOF, highlighting the potential need for dose adjustments in clinical settings, particularly for RA patients with comorbidities, to mitigate DDI risks. Further human studies are essential to validate these findings and establish safe co-administration guidelines to optimize the therapeutic outcomes of these drugs.

## Figures and Tables

**Table 1:** PK parameters (median ± interquartile range) of TOF in rats (with or without coadministration with RTV) derived using NCA.

Parameter	Control (TOF Only)	RTV 10 mg/kg	RTV 20 mg/kg	Significance (*p*)
AUCinf (ng∙h/mL)	2718 [2468–3200]	8477 6549–9367]	15,980 [15,206–16,789]	<0.001
AUCinf/Dose (ng∙h/mL per mg/kg RTV)	-	848 [655–937]	799 [760–839]	-
Cmax (ng/mL)	1362 [1263–1410]	2250 [1787–2531]	3899 [3589–4263]	<0.001
Cmax/Dose (ng/mL per mg/kg RTV)	-	225 [179–253]	195 [179–213]	-
Tmax (h)	0.25 [0.25–0.5]	0.5 [0.44–1.0]	1.0 [1.0–1.0]	<0.01
t1/2 (h)	1.21 [1.14–1.49]	1.0 [0.92–1.18]	0.87 [0.72–1.01]	>0.05
CL (L/h/kg)	3.57 [2.65–4.49]	1.65 [1.19–2.11]	0.66 [0.60- 0.73]	<0.001

AUC_inf_, the area under the curve from 0 to infinity. AUC_inf_/Dose (ng∙h/mL per mg/kg RTV), AUC normalized by ritonavir dose. C_max_, the maximum plasma concentration. C_max_/Dose (ng/mL per mg/kg RTV); C_max_ normalized by ritonavir dose. T_max_, time to reach the maximum concentration. t_1/2_, half-life, CL, clearance.

## Data Availability

The datasets used in this study are available from the corresponding author upon reasonable request.

## Supplementary Materials

The following supporting information can be downloaded at: https://www.mdpi.com/article/10.3390/ph18101561/s1, Figure S1: Representative chromatograms of TOF in rat plasma. (a) LLOQ (1 ng/mL), (b) study sample near Cmax, and (c) IS sample; Table S1: Linearity of TOF calibration standards in rat plasma (weighted 1/x regression); Table S2: Intraday and Interday precision and accuracy % of measured concentrations of analyte.
