# Supplementary material for: The Effects of Ritonavir on the Pharmacokinetics of Tofacitinib in Rats
Source: Pharmaceuticals (Basel). 2025 Oct 16;18(10):1561. doi: 10.3390/ph18101561 (PMC12567268; doi:10.3390/ph18101561)
Supplement: Supplementary file 1 [file pharmaceuticals-18-01561-s001.zip › pharmaceuticals-3916495-supplementary.pdf]

Supplementary Materials

1. Instrument, chromatography, and mass spectrometric conditions

1.1. Instrument

Plasma concentrations of tofacitinib were measured using the high-performance liquid chromatography (1200 series HPLC; Agilent, Santa Clara, CA, USA) coupled to mass spectrometry (Qtrap 4000; Sciex, Framingham, MA, USA). Tofacitinib (TOF) and domperidone (internal standard, IS) were separated by YMC-Triart C18 column (50 × 3.0 mm, 3μm, YMC, Kyoto, Japan), maintained at 20 °C. The system was run in gradient mode with mobile phase A (0.1 % formic acid in water) and mobile phase B (0.1 % formic acid in acetonitrile) at a flow rate of 0.4 mL/min. Mobile phases were degassed ultrasonically for 20 minutes prior to use. The injection volume was 5 μL. The gradient program for mobile phase B was as follows: maintained at 5% for 0.5 min, linearly increased to 95% over 1.5 min and held until 3 min, decreased to 90% over 3.5 min and maintained until 6 min, then gradually returned to 5% over 7 min and equilibrated under initial conditions until 11 min.

1.2. Mass spectrometric conditions

The mass spectrometry analysis was conducted with an electrospray ionization probe in the positive ion mode. The ion spray voltage was 5500 V and the source temperature was 500 °C. For TOF, the transition was m/z 313.2 → 149.2 with DP 101 V, CE 43 V, and CXP 28 V. For IS, the transition was m/z 426.3 → 175.1 with DP 126 V, CE 39 V, and CXP 10 V.

2. Sample preparation

2.1. Calibration standards

Calibration standard samples were prepared by spiking blank rat plasma (18 μL) with 2 μL of TOF working solutions to yield final concentrations of 1, 10, 25, 100, 250, 1000, 2500, and 5000 ng/mL. Each standard was further mixed with 400 μL of acetonitrile containing the internal standard (25 ng/mL). After vortex mixing (10 min) and centrifugation (15,000 g, 10 min, 4 °C), 50 μL of the supernatant was transferred for LC–MS/MS analysis.

2.2. Quality control (QC) sample

QC samples were prepared using the same procedure as the calibration standards at three concentration levels: LQC (2.5 ng/mL) MQC (1500 ng/mL), HQC (4000 ng/mL). Three replicates were prepared at each level from separate primary and working stock solutions and analyzed alongside freshly prepared calibration curves to assess accuracy and precision.

3. Calibration curve and QC validation

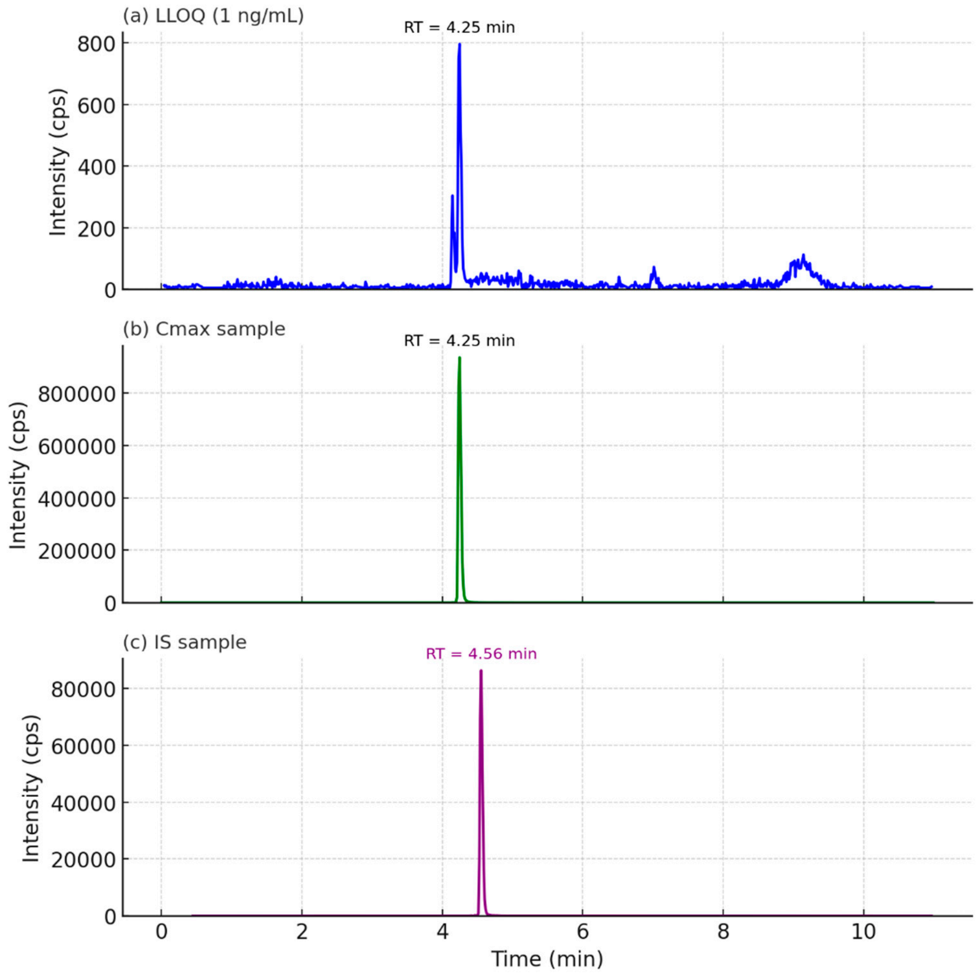

Figure S1. Representative chromatograms of TOF in rat plasma. (a) LLOQ (1 ng/mL), (b) study sample near Cmax, and (c) IS sample.

Table S1 Linearity of TOF calibration standards in rat plasma (weighted 1/x regression)

Nominal Conc. (ng/mL)	Measured Conc. (ng/mL)	Precision (CV %)	Accuracy %
1	1.07	2.44	107
10	10.8	5.74	108
25	28.7	1.91	115
100	109	5.31	109
250	290	4.41	116
1000	1080	3.83	108
2500	2580	4.23	103
5000	4590	0.91	92

Regression equation: $y = 0.00372\ x + 0.00581$ ($R^2 > 0.99$)

- The calibration curve was constructed using a weighted (1/x) linear regression

Table S2 Intraday and Interday precision and accuracy % of measured concentrations of analyte

Nominal Conc. (ng/mL)	Intra-Day Measured Conc. (Mean± SD)	Precision (CV %)	Accuracy %	Inter-Day Measured Conc. (Mean± SD)	Precision (CV %)	Accuracy %
LQC	2.71 ± 0.15	5.5	108.5	2.79 ± 0.17	6.1	116.6
MQC	1520 ± 88.9	5.9	101.3	1530 ± 78.3	5.1	102.0
HQC	3750 ± 118	3.2	93.8	3710 ± 109.6	3.0	92.8

- All QC levels met the acceptance criteria of accuracy (within ±15% of nominal; ±20% for LLOQ) and precision (CV ≤15%; ≤20% for LLOQ), as recommended by FDA/EMA bioanalytical method validation guidance.
